# CD117 immunoexpression in canine mast cell tumours: correlations with pathological variables and proliferation markers

**DOI:** 10.1186/1746-6148-3-19

**Published:** 2007-08-21

**Authors:** Rui M Gil da Costa, Eduarda Matos, Alexandra Rema, Célia Lopes, Maria A Pires, Fátima Gärtner

**Affiliations:** 1 Instituto de Ciências Biomédicas de Abel Salazar (ICBAS), University of Porto, Porto, Portugal; 2 Veterinary Sciences Department, University of Trás-os-Montes and Alto Douro, Vila Real, Portugal; 3 Institute of Molecular Pathology and Immunology of the University of Porto (IPATIMUP), Porto, Portugal

## Abstract

**Background:**

Cutaneous mast cell tumours are one of the most common neoplasms in dogs and show a highly variable biologic behaviour. Several prognosis tools have been proposed for canine mast cell tumours, including histological grading and cell proliferation markers. CD117 is a receptor tyrosine kinase thought to play a key role in human and canine mast cell neoplasms. Normal (membrane-associated) and aberrant (cytoplasmic, focal or diffuse) CD117 immunoexpression patterns have been identified in canine mast cell tumours. Cytoplasmic CD117 expression has been found to correlate with higher histological grade and with a worsened post-surgical prognosis. This study addresses the role of CD117 in canine mast cell tumours by studying the correlations between CD117 immunoexpression patterns, two proliferation markers (Ki67 and AgNORs) histological grade, and several other pathological variables.

**Results:**

Highly significant (p < 0,001) correlations were found between CD117 immunostaining patterns and histological grade, cell proliferation markers (Ki67, AgNORs) and tumoral necrosis. Highly significant (p < 0,001) correlations were also established between the two cellular proliferation markers and histological grade, tumour necrosis and epidermal ulceration. A significant correlation (p = 0.035) was observed between CD117 expression patterns and epidermal ulceration. No differences were observed between focal and diffuse cytoplasmic CD117 staining patterns concerning any of the variables studied.

**Conclusion:**

These findings highlight the key role of CD117 in the biopathology of canine MCTs and confirm the relationship between aberrant CD117 expression and increased cell proliferation and higher histological grade. Further studies are needed to unravel the cellular mechanisms underlying focal and diffuse cytoplasmic CD117 staining patterns, and their respective biopathologic relevance.

## Background

While remaining infrequent in human beings, cutaneous mast cell tumours (MCTs) are one of the most common tumours in dogs, accounting for about 6% of all tumours and 13% of all skin tumours of the dog [1]. Thus, canine MCTs have attracted increasing attention in recent years, as spontaneous models for studying mast cell neoplastic disorders and developing new targeted chemotherapeutic drugs [2-4]. The biological behaviour of canine MCTs can vary widely and is often difficult to predict [1]. Histological grading [5] is widely used for prognosis analysis [6], but its adequacy remains debatable [7]. According to the internationally adopted system, MCTs are usually graded as well-differentiated (grade I), moderately differentiated (grade II) or poorly differentiated (grade III) tumours. Other prognostic factors have recently been proposed, most notably proliferation markers such as Ki67 (MIB-1) nuclear antigen labelling index and AgNORs mean counts [8-11]. Recent studies have demonstrated both normal (membrane associated) and abnormal (cytoplasmic, focal or diffuse) CD117 immunoexpression patterns in canine MCTs [12-14]. CD117 cytoplasmic expression patterns have been shown to correlate with reduced post-surgical survival [15]. Together with it's ligand (stem cell factor – SCF), also known as Steel factor or KIT ligand, CD117 (also referred as KIT or c-KIT) is a critical transmembrane receptor tyrosine kynase for a number of cell types, including some hematopoietic stem cells, mast cells, melanocytes, and germ cells. Binding of SCF by CD117 leads to receptor dimerization and activation of its tyrosine kynase activity [16]. A number of signal transduction pathways, such as the PI3-kinase and the RAS/Erk pathways have been implicated in mediating CD117 functions in mast cells, including cellular proliferation and differentiation, resistance to apoptosis, mobility and chemotaxis, adhesion to fibronectin and enhancement of serotonin and histamine release [17-19]. CD117 is encoded by the proto-oncogene *c-kit *[16]. Point mutations of this gene have been associated with human mastocytosis [20,21] and other malignancies [22-26] while *c-kit *exon 11 deletions and duplications have been identified in canine MCTs [27-30]. This work aims to study the role of CD117 in canine MCTs by analysing the correlations between CD117 immunoexpression patterns, two proliferation markers (Ki67 and AgNORs), histological grading, and several pathological variables.

## Results

**Figure 1:**
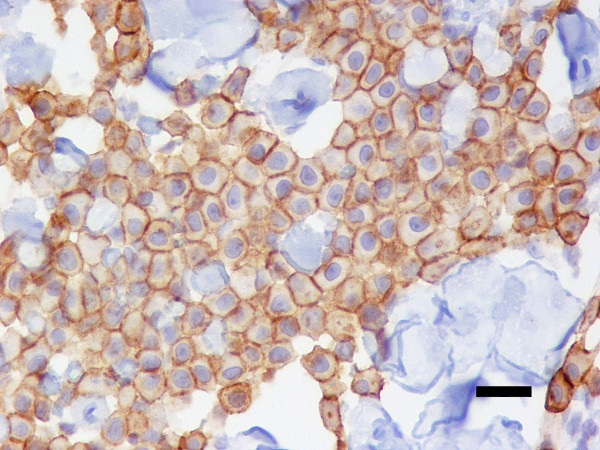
**Membrane-associated CD117 immunostaining**. Canine cutaneous MCT showing membrane-associated immunostaining for anti-CD117 antibodies. ABC 400×. Bar = 20 μm.

**Figure 2:**
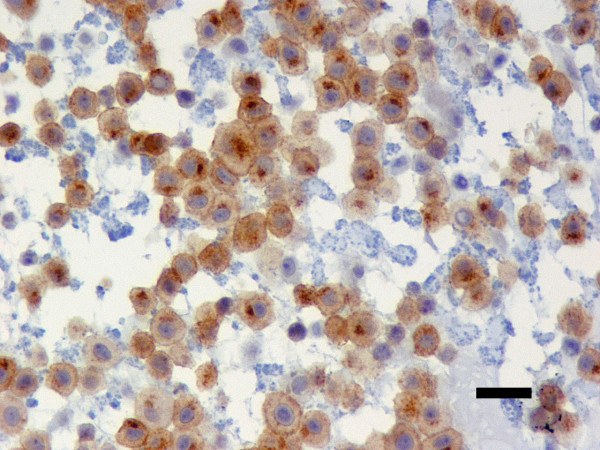
**Focal cytoplasmic (Golgi-like) CD117 immnostaining**. Canine cutaneous MCT showing focal cytoplasmic immunostaining for anti-CD117 antibodies. ABC 400×. Bar = 20 μm.

**Figure 3:**
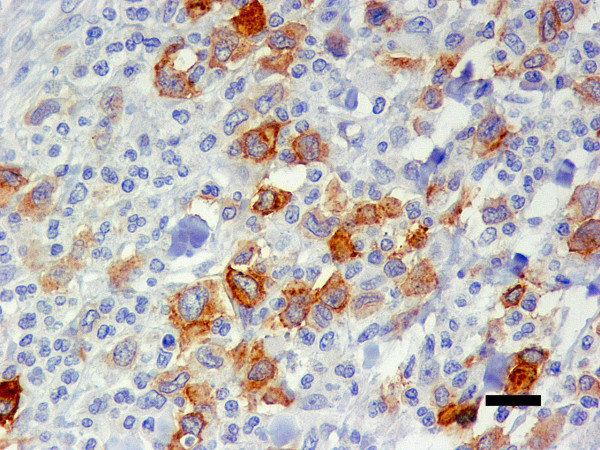
**Diffuse cytoplasmic CD117 immnostaining**. Canine cutaneous MCT showing diffuse cytoplasmic immunostaining for anti-CD117 antibodies. ABC 400×. Bar = 20 μm.

**Figure 4:**
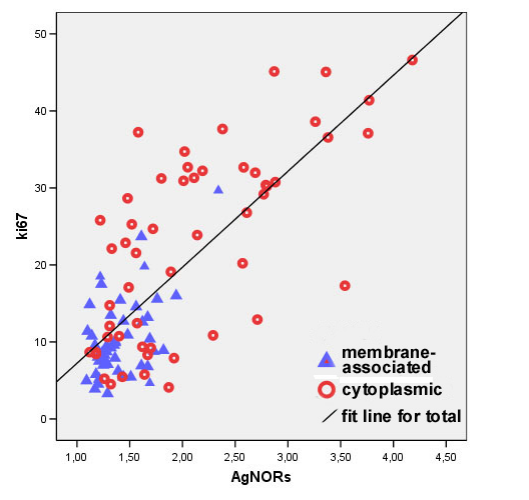
Correlations between Ki67 labelling index, AgNORs mean counts and CD117 staining patterns.

49 (47.6%) tumours presented a membrane-associated CD117 staining pattern (Figure 1), while 46 (44.7%) showed focal cytoplasmic staining (Figure 2) and 8 (7.8%) diffuse cytoplasmic staining (Figure 3). Ki67 labelling index ranged from 3.3 to 46.6, while mean nuclear AgNORs counts ranged from 1.1 to 4.2. Histologically, 35 (34.0%) tumours were grade I, 45 tumours (43.7%) were grade II and 22 tumours (22.3%) were grade III. Highly significant statistical correlations (p < 0,001) were found between CD117 staining patterns and histological grade, presence of necrosis and mitotic index (Table 1). However, no differences were observed between focal and diffuse staining patterns, concerning any of the variables studied. Highly significant statistical correlations (p < 0,001) were also established between Ki67 labelling index and CD117 staining patterns, histological grade, mitotic index and presence of necrosis and ulceration (Table 2) as well as between these variables and AgNORs counts (Table 3). Figure 4 shows a linear correlation between Ki67 labelling index and mean AgNOR counts (R Spearman correlation coefficient 0.639) and the distribution of CD117 staining patterns (membrane-associated versus cytoplasmic) according to these proliferation markers. AgNORs mean counts were significantly higher in MCTs showing cytoplasmic CD117 expression (p < 0,001). A significant correlation was found between CD117 cytoplasmic pattern and epidermal ulceration (p = 0.035). No significant statistical correlation was established between any pathologic variables and the sex, age or breed of the animals, nor with the number (single/multiple) or location (head and neck, trunk, limbs, perineal) of the lesions.

**Table 1:** CD117 immunoexpression in canine MCTs (membrane-associated versus cytoplasmic staining): correlations with multiple pathologic variables

Pathologic variables	CD117 expression pattern		P value
		
	membrane-associated	cytoplasmic	
Histological grade			
I	24 (49.0%)	11 (20.4%)	p < 0,001
II	22 (44.0%)	23 (42.6%)	
III	3 (6.1%)	20 (37.0%)	
Necrosis			
No	44 (89.8%)	29 (53.7%)	p < 0,001
Yes	5 (10.2%)	25 (46.3%)	
Growth pattern			
well circumscribed	7 (14.3%)	5 (9.3%)	P = 0,427
invasive	42 (85.7%)	49 (90.7%)	(NS)
Ulceration			
No	40 (81.6%)	34 (63.0%)	P = 0,035
yes	9 (18.4%)	20 (37.0%)	
Mitosis (per high power field)			
0	37 (75.5%)	22 (40.7%)	
1	11 (22.4%)	8 (14.8%)	p < 0,001
2	1 (2.0%)	10 (18.5%)	
≥ 3	0 (0.0%)	14 (25.9%)	

NS – non-significant

**Table 2:** Ki67 labelling index in canine MCTs: correlations with multiple pathologic variables

Pathologic variables	Ki67 labelling index				(p value)
	**median**	**min**.	**max**.	**IR**	
Histological grade					
I	7.1	3.3	14.9	3.2	
II	13.3	3,9	32.7	12.9	p < 0,001
III	32.2	15.4	46.6	8.5	
Necrosis					
No	9.7	3.3	38.6	10.2	p < 0,001
Yes	27.7	6.2	46.6	20.6	
Growth pattern					
well circumscribed	8.2	4.1	46.6	13.8	P = 0,158
Invasive	12.7	3.3	45.1	17.4	NS
Ulceration					
No	9.8	3.3	46.6	10.8	p < 0,001
Yes	29.4	4.5	45.1	19.4	
Mitoses (per high power field)					
0	8.6	3.3	29.7	4.7	
1	16.0	9.3	30.8	11.3	p < 0,001
2	29.2	15.4	45.1	15.1	
≥ 3	33.7	30.9	46.6	7.5	
CD117 staining pattern					
membrane-associated	9.2	3.3	29.7	6.0	
cytoplasmic focal	23.9	4.1	46.6	21.7	p < 0,001
cytoplasmic diffuse	19.1	5.8	45.1	21.6	

IR – interquartile range, NS – non-significant.

**Table 3:** AgNORs mean counts in canine MCTs: correlations with multiple pathologic variables

Pathological variables	AgNORs mean counts				(p value)
	**Median**	**min**.	**max**.	**IR**	
Histological grade					
I	1.3	1.1	2.3	0.4	
II	1.5	1.1	2.9	0.5	p < 0.001
III	2.4	1.2	4.2	1.6	
Necrosis					
No	1.4	1.1	3.8	0.5	p < 0.001
yes	1.9	1.2	4.2	1.3	
Growth pattern					
well circumscribed	1.3	1.2	4.2	0.6	P = 0.245
invasive	1.6	1.1	3.8	0.7	(NS)
Ulceration					
no	1.4	1.1	4.2	0.5	p < 0.001
yes	1.8	1,2	3.8	1.3	
Mitoses (per high power field)					
0	1.4	1.1	2.7	0.4	
1	1.6	1.2	2.9	0.4	p < 0.001
2	2.3	1.3	3.5	1.3	
≥ 3	2.6	1.8	4.2	1.4	
CD117 staining					
membrane-associated	1.3	1.1	2.3	0.4	
cytoplasmic focal	1.9	1.1	4.2	1.2	p < 0,001
cytoplasmic diffuse	1.6	1.2	3.4	1.3	

IR – interquartile range, NS – non-significant.

## Discussion

The predominance of the Boxer breed among the animals studied reflects a well-known breed predisposition for canine MCTs. However, no significant correlations were found between the breed of the animals and any other of the variables now studied, and the significance of breed regarding the biopathology of MCTs remains unclear. The linear correlation observed between Ki67 labelling index and mean AgNOR counts validates the results of each technique and allows for confirmation of differences in cellular proliferation by two independent methods. The Ki67 labelling index increases in a step-wise way from histological grade I to III, but there is considerable overlapping of both AgNORs and Ki67 values between histological grades. Results have highlighted a strong correlation between cytoplasmic (altered) CD117 immunoexpression and increased cell proliferation and higher histological grade (itself partly based on mitotic index) when compared with the normal, membrane-associated expression pattern. Two distinct patterns for CD117 cytoplasmic staining (a focal, paranuclear, sometimes referred to as Golgi-like pattern and a diffuse pattern) have been described [12-15]. In this study, no significant differences were found between focal and diffuse cytoplasmic CD117 staining, concerning any of the variables studied. This suggests that focal and diffuse cytoplasmic staining may reflect similar cellular changes and, possibly, a progressive cytoplasmic accumulation of CD117. Additional studies are needed to elucidate the biopathologic relevance of these expression patterns as well as the corresponding underlying cellular mechanisms. *C-kit *mutations have been shown to induce ligand-independent (constitutive) CD117 phosphorylation and activation in human neoplasms, both by impairing the regulatory functions of the juxtamembrane domain and by directly targeting the kinase domain [31]. Such mutations are likely to be the cause of increased cell proliferation in MCTs showing cytoplasmic CD117 expression. It is interesting to speculate that mutations causing constitutive CD117 phosphorylation may also collide with the intracellular traffic of CD117 and cause the molecule to accumulate in cellular organelles, such as the Golgi apparatus or the endoplasmic reticulum. *C-kit *mutations have been shown to correlate with altered CD117 expression, though mutations weren't present in all MCTs with aberrant CD117 expression [32]. By elucidating the precise cellular location of cytoplasmic CD117 accumulations, especially in those lesions presenting a "Golgi-like" pattern, a deeper insight into the role of this molecule in tumorigenesis may perhaps be gained. Results suggest that translocation of CD117 into the cytoplasm correlates with activation of CD117-mediated cell proliferation. Although increased cell proliferation in itself is insufficient for neoplastic transformation, it may contribute to the malignant phenotype by increasing the risk of spontaneous mutations. Some human neoplasms have been demonstrated to present an autocrine loop in which neoplastic cells express both SCF and CD117 [33], thus securing permanent growth stimulation. This hypothesis has not been investigated in the case of canine MCTs and should be addressed in future studies. The correlation observed between tumoral necrosis and cytoplasmic CD117 expression (Table 1) may reflect increased cellular proliferation, insufficiently accompanied by angiogenesis, as suggested by the correlation observed between necrosis and higher Ki67 labelling index (Table 2). On the other hand, the correlation between the CD117 cytoplasmic pattern and epidermal ulceration may be due to a CD117-mediated enhancement of histamine and serotonine release and consequent pruritus and self-induced trauma. CD117 might also be thought to play a role in tumoral progression, as suggested by the correlation of cytoplasmic CD117 staining and higher histological grade, by facilitating neoplastic cell mobility and binding of fibronectin. The tumoral growth pattern (well circumscribed *versus *invasive) and the clinical variables studied (race, sex, age, tumour number and location) have shown no correlations with any of the pathological variables studied. The available survival data doesn't allow for conclusions as to which of the factors now studied is more suitable for prognostic analysis.

## Conclusion

Cytoplasmic (altered) expression of CD117 correlates with increased cellular proliferation, as assessed by both Ki67 labelling index and by AgNORs mean counts. This is in accordance with the known functions of CD117 as a growth factor receptor and is probably associated with a *c-kit *mutation. Moreover, cytoplasmic CD117 expression also correlates with increased histological grade, tumour necrosis and epidermal ulceration. No differences were observed between focal and diffuse cytoplasmic staining patterns, suggesting that these represent similar cellular changes, or perhaps a progressive process of cytoplasmic CD117 accumulation. Our future aims are to look for possible mutations in the *c-kit *gene in canine MCCs.

## Methods

### Samples collection and processing

103 MCTs surgically removed from 67 dogs, between 2000 and 2006, were sent for examination at the ICBAS-UP Veterinary Pathology laboratory. The animals comprised 30 males and 37 females, with ages ranging between 2 and 20 years and a mean age of 7.3 years. 33 (49.25%) animals were of the Boxer breed, 18 (26.87%) of mixed breed and 16 (23.88%) of other breeds (with 3 or less animals of each breed). All nodules were considered as primary MCTs, even when occurring in a multiple pattern. Samples were fixed in 10% buffered formallin and paraffin-embedded. Thin serial sections were obtained for each sample and used for routine haematoxylin-eosin (H&E) staining, AgNOR staining and immunohistochemical detection of CD117 and Ki67. 3 μm-thick sections were used for all techniques except AgNORs (4 μm).

### Histological evaluation

Histological grading was performed on H&E-stained slides, following Patnaik's system [5], by two independent pathologists (RMGC and FG). When grading differed, decision was taken by consensus. The number of mitotic figures per high power field and other pathologic changes, such as tumoral necrosis and epidermal ulceration were also noted.

### AgNORs staining

AgNOR staining was performed as previously described [34]. The staining solution was freshly prepared for each experiment, from a 2% gelatin solution (Merck) and 50% silver nitrate (Merck), in a 1:2 ratio. Slides were immersed in the staining solution under conditions of reduced light for 45 minutes and washed in deionised water. Silver deposits were fixed in a 5% sodium thiosulfide (Merck) solution. Slides were then counterstained with light green (Merck) dehydrated, cleared and mounted in synthetic mounting medium (Entellan, Merck). Preparations were then examined on a light microscope, using a 100× objective, and the mean AgNOR count per nucleus was determined in 100 neoplastic mast cells, as previously described [35]. AgNORs were counted excluding areas of necrosis and inflammation.

### Immunohistochemistry

Monoclonal antibodies against Ki67 (MIB-1, Dakopatts, Denmark) and polyclonal antibodies against CD117 (DakoCytomation, USA) were employed, using the avidin-biotin-peroxidase (ABC) method. Heat-induced antigen retrieval (HIER) was performed: for Ki67, slides were incubated for 30 minutes in a commercial antigen retrieval solution (Dako), at 100°C in a water bath. For CD117, slides were incubated in a 10 mM citrate buffer (pH = 6.0) in a steamer, for 2 minutes. Endogenous peroxidase activity was blocked by immersing slides in methanol containing 3% hydrogen peroxide for 10 minutes. Anti-Ki67 and anti-CD117 antibodies were diluted at 1:50 and 1:450 in 5% bovine serum albumin, respectively. Slides were incubated with antibodies overnight at 4°C. Human gastrointestinal stromal tumours (GISTs) were used as positive controls for CD117 staining. Detection was performed using 3,3'-diaminobenzidine substrate (Dako). Sections were then counterstained with Mayer's haematoxylin, dehydrated, cleared and mounted in Entellan mounting medium (Merck). Slides were evaluated under light microscopy. The KI67 index was determined in areas with high labelling immunoreactivity, excluding areas of necrosis and inflammation, per 1000 cells, as previously described [36]. For CD117, three staining patterns were recognized: a membrane-associated pattern with little to none cytoplasmic staining, a focal (paranuclear or Golgi-like) cytoplasmic pattern, with only occasional minor membrane staining and a diffuse cytoplasmic pattern [12-15].

### Statistical analysis

Nonparametric analysis was conducted, using SPSS 14.0, with a significance level of 5% and bilateral tests. Pearson's independent chi-squared test was used to assess the correlations between CD117 immunostaining patterns and several clinical and pathological variables. Kruskal-Wallis test was used to assess differences between Ki67 median values and AgNORs mean counts of different variables. A Spearman's correlation was calculated between Ki67 labelling index and AgNORs mean counts.

## Authors' contributions

RMGC participated in conceiving and designing the study, diagnosed and graded the tumours, interpreted histochemical and immunohistochemical assays and drafted the manuscript; EM performed the statistical analysis, CL and AR carried out the histochemical and immunohistochemical assays; FG conceived and designed the study, participated in diagnosing and grading the tumours and in interpreting the results. MAP participated in designing the study and interpreting the results. All authors read and approved the final manuscript.
